# Plasma Electrolytic Oxidation of Titanium in Ni and Cu Hydroxide Suspensions towards Preparation of Electrocatalysts for Urea Oxidation

**DOI:** 10.3390/ma16062191

**Published:** 2023-03-09

**Authors:** Marta Wala, Dorota Łubiarz, Natalia Waloszczyk, Wojciech Simka

**Affiliations:** Faculty of Chemistry, Silesian University of Technology, Krzywoustego Str. 6, 44-100 Gliwice, Poland

**Keywords:** plasma electrolytic oxidation, black TiO_2_, urea oxidation, electrocatalyst

## Abstract

The increasing climate crisis requires an improvement in renewable energy technologies. One of them are fuel cells, devices that are capable of generating electricity directly from the chemical reaction that is taking place inside of them. Despite the advantages of these solutions, a lack of the appropriate materials is holding them back from commercialization. This research shows preliminary results from a simple way to prepare black TiO_2_ coatings, doped with Cu or Ni using the plasma electrolytic oxidation process, which can be used as anodes in urea-fueled fuel cells. They show activity toward urea oxidation, with a maximum current density of 130 μA cm^−2^ (@1 V vs. Hg|HgO) observed for Cu-enhanced TiO_2_ and low potential of only 0.742 V (Vs Hg|HgO) required for 50 μA cm^−2^ for Ni-enhanced TiO_2_. These results demonstrate how the PEO process can be used for the preparation of TiO_2_-based doped materials with electrocatalytic properties toward urea electrooxidation.

## 1. Introduction

The increasing climate crisis and the growing tension around fossil fuels show that new energy sources are needed. Among alternative power sources, fuel cells are devices capable of generating electricity directly from the electrochemical reaction taking place inside them. As fuel, a variety of compounds can be used, from fossil fuels to hydrogen or simple organic molecules, such as methanol [1,2,3,4], ethanol [1,4,5,6] or urea [7,8].

Urea as a molecule deserves special attention due to its presence in the environment in huge quantities. Every mammal excretes urea with its urine and huge amounts of urea are released into the environment from the chemical industry and agriculture [9,10]. Its oxidation is a six-electron reaction, which makes it pretty energetic, but at the same time, the transport of that 6 e^−^ makes the urea oxidation reaction (UOR) sluggish [7,10,11]. To improve its kinetic variety, many materials can be used as an electrocatalyst, mainly based on nickel, due to its activity in the oxidation of simple organic molecules [11,12,13]. Another metal that is active towards UOR is copper, which, like nickel, is a cheap and available material, but is less harmful due to its non-toxicity [11,13,14].

Plasma electrolytic oxidation (PEO) is an electrochemical technique of surface modification. The modified detail is anodically polarised in a proper electrolyte, resulting in the growth of the oxide layer on its surface. During this process, the resistivity of the layer increases because of the dielectric properties of the growing layer. After the thickness of the oxide layer reaches its critical value, if the voltage is high enough to exceed the dielectric breakdown voltage, dielectric breakdowns occur on the surface of the modified element. These breakdowns modify the oxide layer, making it thicker and more porous. Due to the rapidity of the process, the electrolyte ingredients can be easily incorporated into the growing layer, showing good potential for using this method for the preparation of catalytic systems [15,16,17,18,19].

Additionally, the PEO process leads to the formation of dielectric, oxide layers, which can also be used as photocatalytic materials, due to the controllable crystalline composition and their solar absorbent properties [20,21,22,23,24,25]. A high proportion of the crystalline phase in the oxide layer on pure titanium with a variable anatase to rutile ratio, which can be controlled by changing the electrolyte, and can be combined with the porosity of the layer to optimise the specific structure to absorb photons [22]. The PEO process used for the preparation of photocatalytic layers has already been proven as a photoelectroactive material; for example, for wastewater treatment it gave better results than just photolysis or photocatalysis [21,25]. Especially interesting results were observed for black PEO layers prepared on a Ti-Al-V alloy [26] and on Ti itself. The black colour is related to the presence of low valent ions of Ti: Ti^3+^ and Ti^2+^, oxides of which are dark purple and black, respectively, and lead to greater absorbance and this increases the potential for the photocatalytic properties of the final material [20].

The reactivity of PEO layers can be easily influenced by changing the composition of the electrolytic bath, which is usually completed by adding soluble ions of dopants, i.e., Ni and Cu, to the electrolytic bath to prepare catalytic materials for CO to CO_2_ oxidation [27,28,29], photoactive materials [26,30,31], biomaterials with possible antibacterial properties [32,33] or even catalysts for desulphurization and denitrification [34]. Other methods of doping of PEO layers are the impregnation of the coating with the soluble salt of the dopant, followed by drying and air annealing (500–1050 °C) [29,35,36,37,38,39,40,41,42], the hydrothermal route [43,44], electrodeposition [45,46], the sol-gel method [47], electroless plating [48], the solid-state reaction method [49], the alcohol-thermal method [50], reactive magnetron co-sputtering [51], dip coating [52] or even the coating of different materials with a TiO_2_ paste [53]. A different approach is the preparation of an electrolytic bath in suspension [18,54,55,56]. Such a process has many advantages, which is that the oxides of doped metals that are built into the layer during the PEO process tend to be more reactive after they are added to the layer than organic, metallic, carbide, or nitrate particles [57].

For these reasons, we decided to use the PEO process to incorporate Ni and Cu into the TiO_2_ layer on a Ti substrate to generate electrocatalytic materials for urea oxidation. To prepare the black coating, we have used a silicate-rich electrolyte, to which we added powders of nickel or copper hydroxides, which have not been described in the literature before, to incorporate them in the forms of oxides and/or hydroxides with potential electrocatalytic properties for urea oxidation.

## 2. Materials and Methods

Titanium grade 2 samples (Bimo Tech Co., Wrocław, Poland) with a 0.5 cm height and 1 cm diameter were prepared for the PEO process by grinding with 240 grade SiC paper and degreasing in isopropanol for 5 min using ultrasound. To ensure that only one site of the sample had contact with the electrolyte samples, the samples were secured with a silicone tube.

To prepare the reference sample, the PEO process was carried out in solution, the compositions of which are presented in Table 1. To incorporate nickel and copper into the PEO oxide layer, their dried and milled hydroxides, with grain sizes smaller than 315 μm, were added to the electrolytic bath with different molar concentrations, shown in Table 1. Metal hydroxides were synthesized by mixing metal salt solutions with an excess of sodium hydroxide solution. Formed precipitations were left for a week to age and then they were filtered using a vacuum pump.

The samples were prepared using a DC power supply (PWR800H, Kikusui, Kyoto, Japan) with a current density of 150 mA cm^−2^ and a limiting voltage of 200 V. Due to the high resistivity of the electrolytic bath in the case of the samples of Cu II, 200 V was not possible to achieve, which is why they were prepared with a different limiting voltage, with a value of 180 V. A stainless steel plate was used as a cathode. To ensure that other samples would not be contaminated with deposited metals, the cathode was ground between the different metal hydroxide series. The electrolytic cell was equipped with a cooling mantle, in which polyethylene glycol was circulated to absorb the process heat. The temperature of the cooling solution was kept at 10 °C by cryostat. Stirring of the electrolyte was performed with a magnetic stirrer. After the PEO process, the samples were washed with deionised water and dried in the exicator.

The morphology and elemental composition of the samples were examined using the Phenom Pro-X scanning electron microscope equipped with an X-ray energy dispersive spectroscope unit (SEM-EDX) (ThermoFischer Scientific, Watham, MA, USA); accelerating voltage—15 kV).

The cross sections of the samples were prepared by inclusion in the epoxy resin, followed by its grinding using SiC polishing papers with a grade from 60 to 1000, followed by polishing using diamond suspension with a 1 μm diamond diameter. Both grinding and polishing were performed using the automatic polisher Forcipol 202 (Metkon Instruments, Bursa, Turkey). The prepared cross sections were examined using the SEM-EDX Phenom Pro-X microscope (ThermoFischer Scientific, Watham, MA, USA).

The coating thickness was analysed using the SEM images of the cross sections. Measuring was performed using the GIMP graphic program, with measurements at 50 points, every 5 μm, calculating the average thickness and its standard deviation.

Surface roughness was examined using the profilometer (SJ-301, Mitutoyo, Kawasaki, Japan). It has been characterised as two parameters: *R_a_* and *R_z_*.

*R_a_* is the arithmetic mean of the sum of the surface profiles for a given sample, according to the following Equation (1):

(1) $$R_{a}=\frac{1}{l}\int_{0}^{1}\left|Z\left(x\right)\right|dx$$

where *l* is the elementary length in the x-direction (average line) used to determine the roughness of the surface profile and *|Z*(*x*)*|* is the absolute ordinate value inside the elementary measuring length.

*R_z_* is the roughness height defined by 10 profile points, calculated using Equation (2):

(2) $$R_{z}=\frac{R_{2}+R_{4}+R_{6}+R_{8}+R_{10}}{5}+\frac{\left|R_{1}+R_{3}+R_{5}+R_{7}+R_{9}\right|}{5}$$

where *R*_2,4,6,8,10_ are the five highest bulges in the elementary length in the x direction (average line), and *R*_1,3,5,7,9_ are the five deepest recess points in the elementary length in the x direction (average line).

The contact angle of the surface was determined using a drop-shape analysis system (OCA 15EC, DataPhysics Instruments, Filderstadt, Germany) using water as a wet medium, with a drop size of 1 μL.

The electrochemical activity of the prepared layers was examined using the Autolab PGSTS 302N potentiostat (Methrom AG, Herisau, Swithzerland). Measurements were performed in classic 3-electrode system, where the examined samples served as a working electrode, a glassy carbon rod was a counter electrode, and a Hg|HgO electrode, filled with 1 M KOH, was the reference electrode. Before activity examination, each sample was activated via 100 scans in 1 M KOH solution in the voltage range of 0 to 1 V vs. a Hg|HgO electrode with a scan rate of 100 mV s^−1^.

Activity in the 1 M KOH solution was examined using a cyclic voltammetry (CV) scan with the scan rate of 10 mV s^−1^ in the voltage range of 0 to 1 V vs. Hg|HgO. Similarly, the electrocatalytic activity towards urea oxidation was examined in 1 M KOH + 0.15 M urea solution.

## 3. Results

**Figure 1:**
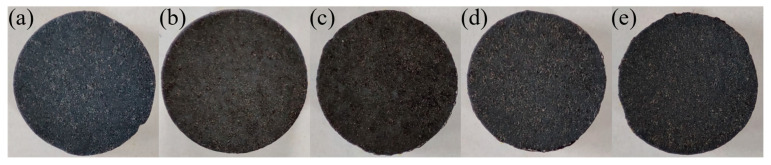
Macro images of prepared samples: (**a**) reference sample, (**b**) Ni I, (**c**) Ni II, (**d**) Cu I, (**e**) Cu II; sample diameter—1 cm.

The conditions of the PEO process in the solution without hydroxides allowed the preparation of the black TiO_2_ layers, with a dark blue undertone, which are presented in Figure 1a. The addition of nickel or copper hydroxides to the electrolytic bath has not strongly influenced the colour of the layers, which gives a high chance of photoactivity in the prepared layers—due to their dark colour they will absorb most of the UV light range, while modification of TiO_2_ with Ni and Cu may increase its photoactivity [45,46,58,59]. The addition of nickel (Figure 1b,c) has resulted in the preparation of slightly more greenish layers, while the addition of copper (Figure 1d,e) has resulted in a change in the layer colour to one that is more grey.

In the literature, black PEO layers are usually obtained for a Ti-6Al-4V alloy [20,26,60]. For their preparation, electrolytes containing Ni [26], Co [26,60], Fe [26,60], W [26] and V [60] are used. However, it has been proven that the preparation of the black PEO coating does not have to be related to the presence of those ions in the layer and that black PEO layers can be prepared on pure titanium in a concentrated silicate solution [20], which is confirmed during our research: the basic PEO bath consisted of only KOH and Na_2_SiO_3_ and the reference sample was still bluish-black, despite the lack of the mentioned ions in the oxidised material and in the electrolytic bath.

**Figure 2:**
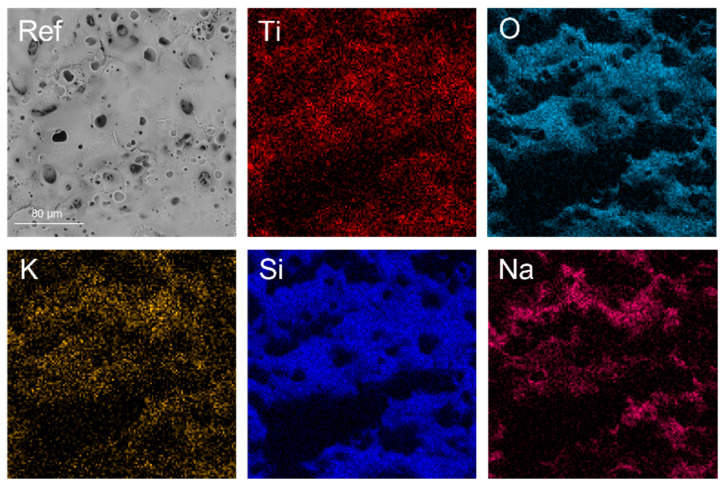
SEM image and EDX mapping of the reference sample.

The microstructure of the prepared layers was examined using a scanning electron microscope. As presented in Figure 2, the PEO process in the electrolyte without suspension has resulted in the formation of a homogenous, porous layer with an even distribution of pores on it entire surface, typical for the PEO process. Micro and mesopores are visible on the surface of the layer, which confirms the preparation of a well–developed surface. Such a morphology should be related to highly active area, which, since catalysis is a surface process, is very beneficial from a potential electrocatalytic use point of view.

EDX analysis, as presented in Figure 2, shows that the reference sample was covered with an oxide layer, containing elements such as titanium, potassium, silicone and sodium, suggesting that the formed oxide layer was made of titanium oxide, coming from the substrate metal and silicone oxide, originating from the electrolytic bath.

**Figure 3:**
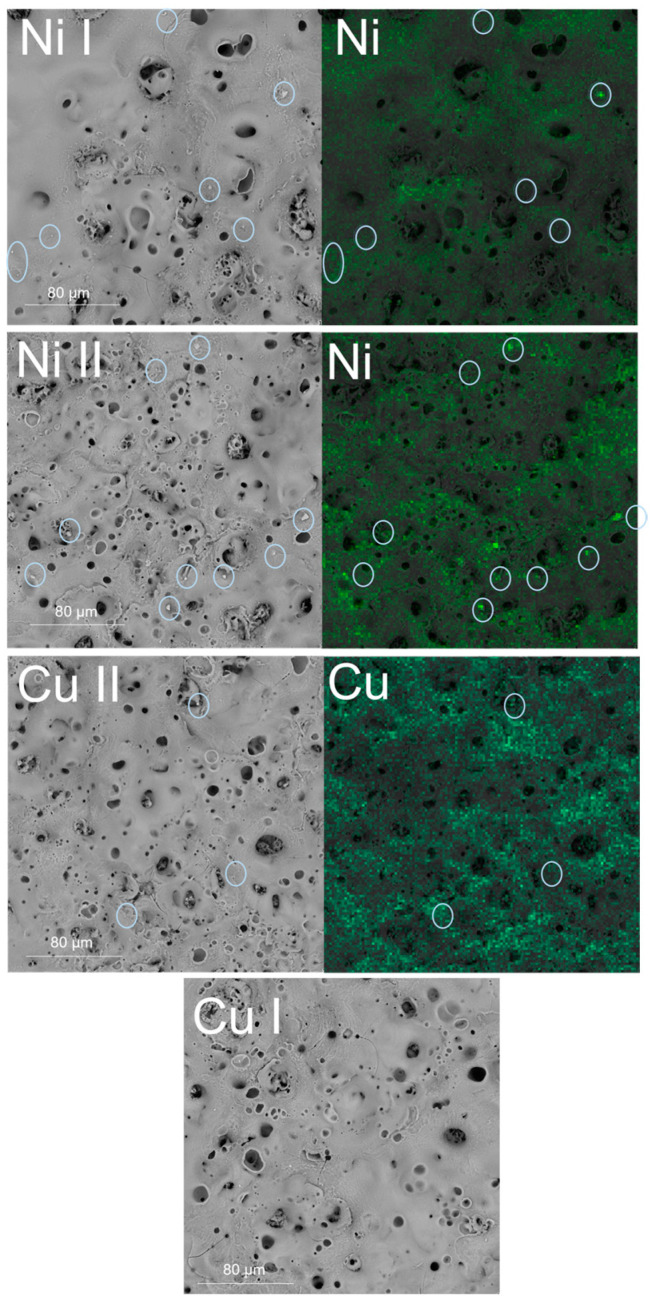
SEM images and EDX mapping of samples anodised in an electrolytic bath with Ni(OH)_2_ and Cu(OH)_2_ suspensions. Blue circles show dispersed particles on the surface of the layer.

As shown in Figure 3, the addition of Ni(OH)_2_ to the electrolytic bath increased the amount of small-diameter pores, agglomerated in clusters, despite using the same current density. During the plasma electrooxidation process, the modified element is positively polarised, which leads to formation of dielectric oxide on its surface. After exceeding the dielectric breakdown voltage due to the formation of a thick oxide layer, the spark ignition takes place, which further modifies the synthesised oxide, leading to the formation of a thicker and porous layer. The presence of the pores is related to the formation of plasma channels, the ionization of the substrate, and melting and solidification of the oxide layer. The ionized metal is then oxidised, and the plasma channel through which it was oxidised is visible as a pore. The size of the pores is related to the power of the electric discharge [15], suggesting that electrolytes containing Ni(OH)_2_ lead to milder discharges, resulting in smaller diameter pores. An increased concentration of Ni(OH)_2_ increased the amount of small pores even more and led to a more homogenous porosity of the layer. Such influence should be very beneficial in terms of the electrochemical activity of the prepared layers, because a higher amount of pores should be related to a higher development of the surface, should lead to a higher amount of active centres, and thus to a higher activity of the layer.

In the case of the electrolytic bath with the addition of copper hydroxide, similar results were obtained. With an increasing Cu(OH)_2_ concentration, the formed oxide layer was covered with a higher amount of small-diameter pores, which means that similarly to Ni(OH)_2_, Cu(OH)_2_ also favoured a higher amount of weaker dielectric breakdown, which led to higher porosity with a lower diameter pore [15]. Such a morphology means that the prepared surface is highly developed and should be characterised with high electrochemical activity. Interestingly, sample Cu II appears to be more porous than sample Cu I, despite using a lower voltage, which should lead to lower porosity [15], suggesting that the presence of copper hydroxide in the electrolytic bath favours the formation of pores on the surface of the oxide layer. On the other hand, during PEO in acidic electrolyte using direct current, the addition of Cu(NO)_3_ to the preparation of porous layers was not possible until the voltage reached 450 V [33]. Using our methodology, the preparation of porous layers with the addition of copper was possible using much lower voltages (below 180 V), which shows that the usage of an alkaline electrolyte with hydroxide suspension favours the preparation of more porous oxide layers during the PEO process.

EDX analysis of the prepared samples shows, as presented in Figure 3, that the addition of nickel hydroxide to the electrolytic bath led to the presence of Ni on the surface of the layer. In the case of a higher amount of added Ni(OH)_2_, the EDX mapping is a brighter green colour, which suggests that the Ni II sample was richer in nickel than Ni I. In the case of Cu(OH)_2_ for the Cu I sample, there was no signal recorded during the EDX mapping, which would be related to the presence of copper on the surface of the layer. Sample Cu II was homogenously covered with copper. Those observations show that the addition of a higher amount of metal hydroxide suspension to the electrolytic bath leads to a higher content of incorporated metal on the surface of the prepared sample.

As presented in Figure 3, small, ball-shaped dispersed particles are visible on the surface of the layers. These formations are formed during the PEO process and could be more enriched in Ni and Cu than the rest of the layer [18,29,61], which is especially visible for the Ni II sample. However, due to the small size of these particles relative to the mapping resolution, it would require further research to fully analyse their chemical composition.

The highest amount of these dispersed particles was observed for the Ni II sample, which suggests that this modification was the most effective in terms of the surface enrichment of Ni. The lowest amount of dispersed particles was observed for Cu II, which might indicate that this sample might be characterised with a low amount of copper present on the surface of the layer in the form of dispersed particles.

During the PEO process, if the metal hydroxides present in the electrolyte are in the form of suspensions and enter the area of electrical breakdown, they undergo dehydration and thermolysis, resulting in the incorporation of their oxides into the layer [15,18,55]. To examine whether the presence of hydroxide suspensions in the electrolytic bath led to the incorporation of nickel and copper into the layer, an EDX analysis was performed. After the addition of nickel and copper hydroxides to the basic electrolytic bath, besides the already mentioned elements, new signals were recorded related to the presence of nickel and copper in the prepared oxide layers, which means that these elements were successfully incorporated into the TiO_2_ layer. However, in the case of the Cu I sample, the signal related to the presence of copper was very weak, which may be related to the low content of this element, or even its absence on the surface of the sample. However, it does not mean that copper was not incorporated into the layer, since it could also be present in deeper parts of the layer.

**Figure 4:**
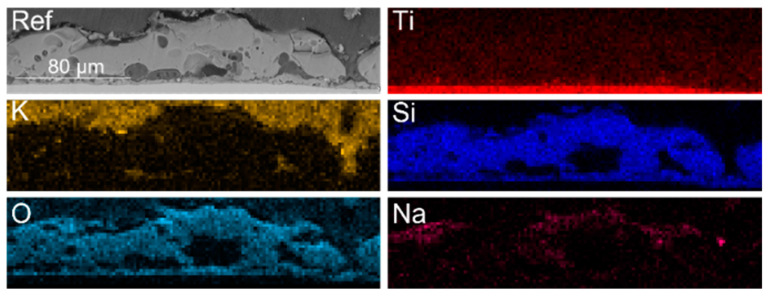
EDX mapping of the cross section of the reference sample.

To examine the inner morphology of the prepared layers and their elemental composition, cross sections of the samples were prepared. As presented in Figure 4, the same elements that were present on the surface of the layer are present in its inner part. The content of potassium, marked above the oxide layer, is related to its presence in the epoxy resin used for cross-section preparation. The prepared reference layer is homogenous, with visible pores in both its outer and inner parts.

**Figure 5:**
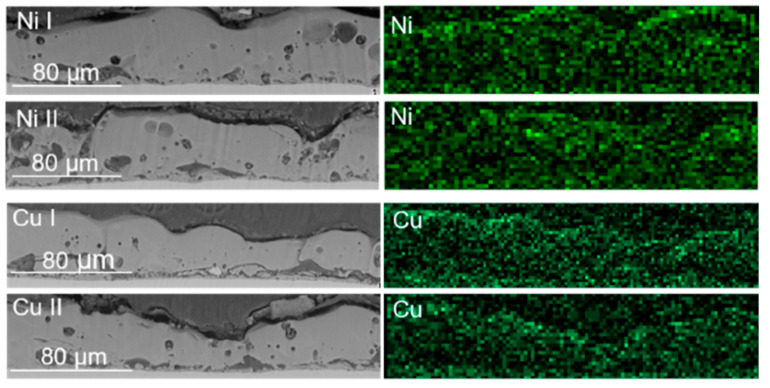
EDX analysis of samples prepared in the electrolytic bath with the addition of nickel and copper hydroxide.

The results of the EDX analysis of the cross sections of samples prepared in the electrolytic bath containing copper and nickel hydroxides are presented in Figure 5. As already proven with the EDX analysis of the layer surface, the addition of nickel hydroxide to the electrolytic bath resulted in the incorporation of this element into the oxide layer. Both prepared layers, Ni I and Ni II, are less porous than the reference layer, with pores still visible both in the inner and outer parts of the layers. In the case of the Ni I sample, the nickel content is more accumulated near the surface of the layer, even though this metal is present in the whole oxide layer. The Ni II sample is characterised by the homogenous presence of nickel across its whole thickness. The Cu I Sample, which showed no presence of copper on its surface, shows the presence of Cu in its deeper layers, similarly to that of the Ni I sample, with a higher copper content closer to the layer surface.

The results show that the addition of metal hydroxides into the electrolytic baths resulted in their being built into the oxide layer. As is presented in Table 2, the difference in the elemental composition of the oxide layers is more visible for the Cu-enriched samples. The addition of a larger amount of Cu(OH)_2_ to the electrolytic bath resulted in a higher copper content on both the surface and inside the layer. This corelation between the atomic content of the layer and the concentration in the electrolyte is not only intuitional but also confirmed by previous research [33]. For samples prepared in the Ni(OH)_2_ suspension, the differences between the atomic content are very small, suggesting that the maximal amount of nickel was incorporated into the layer using the lower concentration of nickel hydroxide, and that its further increase does not influence the layer composition that strongly.

The difference in the amount of incorporated metals is probably caused by their influence on the intensity of the dielectric breakdowns observed during the PEO. As has been mentioned previously, both Ni and Cu lead to fewer energetic sparks, which can result in smaller pore diameters. Additionally, if the PEO process is milder, the amount of ions incorporated from the electrolytic bath into the layer is also lower. Such an influence would explain why the addition of the same molar amount of Ni and Cu hydroxides led to the incorporation of a higher amount of Ni, probably because the influence of copper on the process intensity is much stronger, than in the case of nickel.

The presence of nickel in the layer, probably in the form of NiO, could positively influence the potential photocatalytic properties of the prepared layers [43,45], which will be examined during further research.

Comparing the amount of nickel and copper in the layer with similar methods of TiO_2_ doping during the PEO process (Table 3), the amounts of the incorporated metals are lower than described in the literature for different electrolytic baths. Such a difference might be related to the process conditions, i.e., limiting voltage or current density, which were chosen focusing mostly on the preparation of black TiO_2_ coatings. However, other researchers reported that incorporated metals are present mainly in the outer part of the oxide layer [33], which we have not observed for our layers. The homogeneous incorporation of metallic dopants into the TiO_2_ layer should increase its conductivity and thus positively influence the electrocatalytic properties of the layers, despite the lower atomic percentage of the dopant on the surface of the layer.

**Figure 6:**
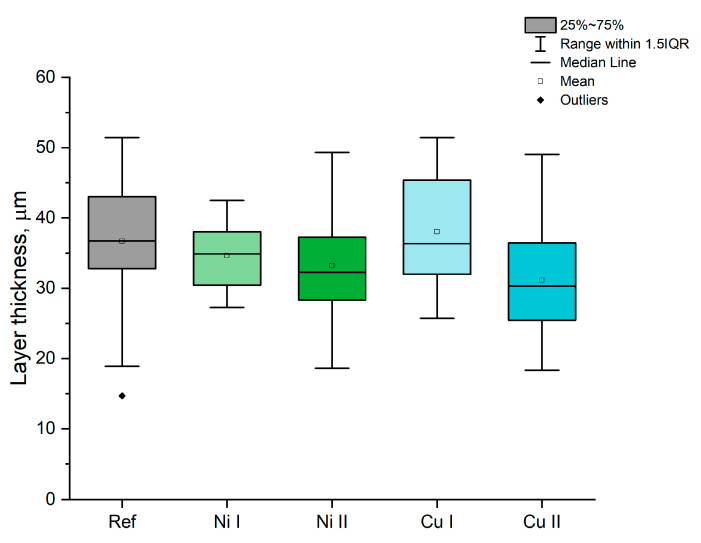
Results of the layer thickness analysis, based on the SEM image of the cross sections of the samples.

On the basis of the SEM images of the layers, the cross-section thickness of prepared layers was examined. As presented in Figure 6, the average thickness of the prepared layer was similar despite different concentrations of the hydroxide suspension. The highest layer thickness median value was observed for the Cu I sample, with a value of 36.4 μm, while the lowest was observed for Cu II with a value of 30.2 μm.

The most homogeneous layer, in terms of its thickness, was observed for the Ni I sample, while the widest range in which the layers thickness values were observed was for the reference sample.

The prepared layers are thicker than the layers prepared in a H_2_SO_4_-based electrolyte described in the literature, which were 2–3 μm thick [21,24,62], with the maximum value of 12 um [24] in a similar voltage range (140–200 V). A similar electrolytic bath, based on silicates, resulted in the preparation of an oxide layer with an average thickness of 13 μm [40], which is almost three times thinner than in our case. Similar values were observed for a phosphate-based solution, with an average thickness of 13.1 μm [63], for an acetic acid-based solution with the addition of nickel acetate, with a value of 12 μm [34], and for a H_3_PO_4_-based solution with the addition of copper nitrate with a value of 27.8 μm [33]. Depending on the process conditions, the oxide layers prepared in PBW (Na_3_PO_4_ + Na_2_B_4_O_7_ + Na_2_WO_4_) electrolytes containing Ni and Cu were thinner (10–14 μm) [28,35], similar (40 μm) [29] or even thicker after the addition of sodium silicate to the electrolytic bath (53.5 μm) [39]. The black coatings prepared on the Ti-6Al-4V alloy were also thinner than our layers, with an average thickness of 24.4 μm [26]. Comparing those results to the ones observed for our layers, the PEO process in alkaline silicates with the addition of Ni and Cu hydroxide suspensions is much more efficient in terms of layer thickness.

Surface roughness is a parameter that can strongly influence the electrochemical activity of materials. Higher roughness is related to the higher development of the surface and thus can be related to a higher active surface of the material. Because the goal was to prepare electrocatalytic material, a higher active surface area would be beneficial because catalysis is a surface process which can be enhanced by increasing the amount of active centres, i.e., by increasing the active surface of the material.

As presented in Table 4, the highest roughness was observed for the Ni II sample, in terms of both parameters: *R_a_* and *R_z_*. The lowest roughness was observed for the Cu I sample, also in terms of both parameters, suggesting that the amount of active centres on the surface of this sample might be the lowest, which may result in the lowest activity among prepared samples. These differences are caused by the influence of the presence of suspensions in the electrolytic bath on the PEO process. As mentioned above, Ni and Cu cause the dielectric breakdowns that occur during the PEO to be less energetic, leading to a smaller pore diameter. Less energetic sparks also do not penetrate the whole oxide layer—to the substrate metal—and thus the formation of large diameter pores, surrounded by ‘pancake structures’ formed by cooling the erupted metal, is limited, and thus the final surface is less rough.

The different influence of copper and nickel hydroxide suspension on the roughness of the final layers might be related to their different nature, which was already shown in the different amounts of incorporated metal present on the surface of the layer and in the deeper parts. In terms of roughness, the addition of nickel hydroxide increased the roughness of the layer; however, the increase in Cu(OH)_2_ content made the layer smoother. Such an observation suggests that the addition of a certain amount of hydroxide suspension to the electrolytic bath would ease up the process and form smoother layers. In the case of Cu(OH)_2_, this concentration was achieved, but in the case of Ni(OH)_2_, an addition of a higher amount would be necessary to observe a similar effect.

When comparing the samples obtained with similar coatings reported in the literature, we observe that our layers are almost two times more rough than the black coating prepared on the Ti6Al4V alloy (*R_a_* of 2.9, *R_z_* of 17.8 μm) [20] and more than the two times rougher than a similar layer prepared in an electrolyte-containing nickel acetate (*R_a_*~2 μm) [26]. However, the plasma electrolytic oxidation of titanium in sodium phosphate resulted in a more rough sample, with an *R_a_* of 17.1 μm [63]. Surface roughness is usually related to the active surface of the layer; more rough surfaces are usually more developed, which is beneficial from the electrocatalytic point of view.

Urea oxidation catalysis is a surface process, which takes place at the phase border between the catalyst and the water-based solution; thus, more hydrophilic surfaces should be characterised with better electrocatalytic properties.

**Figure 7:**
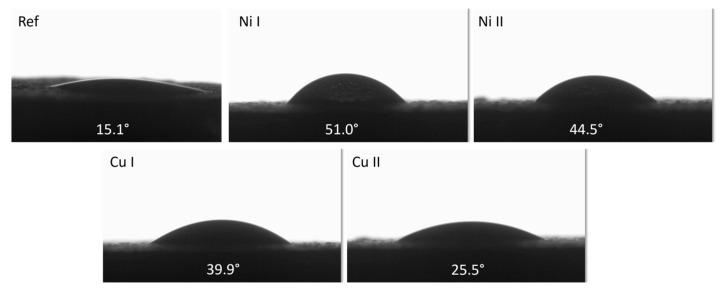
Contact angles and water drop views on prepared samples surface.

All prepared samples show values lower than 90°, which confirms their hydrophilic character (Figure 7). Interestingly, the addition of copper and nickel hydroxides into the electrolytic bath increased the values of the contact angle, making them more hydrophobic than the reference sample. Samples prepared in the presence of nickel hydroxide were more hydrophobic than those prepared in electrolytic baths containing copper hydroxide. Among the samples containing Cu and Ni, the Cu II sample had the lowest contact angle, with a value of 25.5°, while the Ni I sample was characterised by the highest contact angle, with a value of 51.0.

The reference sample had a contact angle two times smaller than that observed for the black coating prepared on the Ti-6Al-4V alloy, which was characterised with a CA of 30° [20]. The addition of hydroxides into the electrolytic bath increased this value, but among the prepared samples, the least rough one, Cu II, still showed a higher hydrophilicity than the alternative black coating.

**Figure 8:**
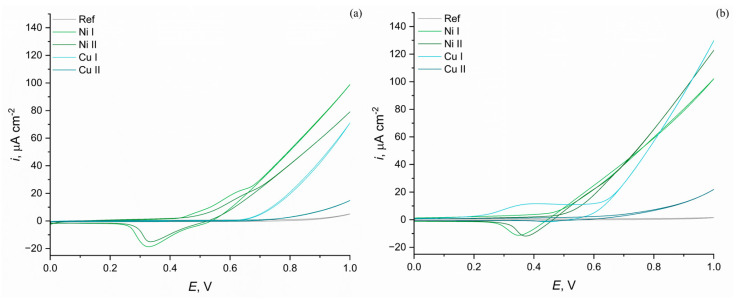
Results of samples’ cyclic voltammetry in (**a**) 1 M KOH solution and (**b**) 1 M KOH + 0.15 M urea solution.

Figure 8 presents the current response of the prepared samples recorded during CV scans, in a 1 M KOH solution with and without the addition of urea. The observed current answers are low due to the dielectric character of the prepared layer, which consists mainly of TiO_2_ and SiO_2_, which are not conductive. However, the addition of nickel and copper hydroxides to the PEO electrolytic baths increased the observed current densities, which is a very promising result in terms of further research. When comparing Figure 8a,b, all samples prepared in electrolytes with hydroxide suspensions gave a higher current response after adding urea to the system, confirming their electrocatalytic properties towards urea oxidation. The lowest current density in both solutions was observed for the reference sample, which showed mainly the background current with no specific activity toward urea oxidation or oxygen evolution.

The addition of copper into the electrolytic bath resulted in an increase in the recorded current densities; interestingly, the activity of the Cu I sample, prepared in a solution containing a lower concentration of copper hydroxide, gave a higher current response in both solutions, despite its lower copper content in the layer.

The addition of nickel into the PEO electrolytic bath gave an electrochemically active layer, with characteristic peaks related to the oxidation of nickel from its II to III valence state during the forward scan, and a reverse reaction during the reverse scan. Interestingly, after the addition of urea, CV cathodic peaks related to the reduction of Ni(III) to Ni (II) are still visible, suggesting that not all of the active centres were used in the reaction [64]. In the case of those samples in the 1 M KOH solution, a higher current response was also recorded for the Ni I sample, prepared in a solution containing less nickel hydroxide; however, after adding urea to the solution, a higher current density was observed for the Ni II sample.

The highest current response in the urea-containing solution was recorded for the Cu I sample, which means that this modification has the highest electrocatalytic activity toward UOR, despite its macrostructure, suggesting that Ni samples should be more active. Such a difference might be related to the better wettability of the Cu samples and the better diffusion transportation of fresh urea portions from the bulk solution to the active centres.

It is agreed that if the material shows a higher current response in the solution containing urea than in the solution without it, it shows electrocatalytic properties toward urea oxidation [64]. Table 5 shows the comparison between the current responses recorded in 1 M KOH with and without the addition of urea for samples prepared via PEO in electrolytes containing metal hydroxide suspensions.

The reference sample shows very low current densities in both solutions, which is related to its dielectric nature, and no significant activity toward OER or UOR. Comparing the results in the presence and in the absence of urea of the Ni I sample, we show very similar current densities at the reverse voltage of 1 V in both solutions. The biggest difference between the current densities at the reverse potentials is visible for the Cu I sample, which is almost doubled after the addition of urea into the solution. The responses of Cu II were the lowest among the Ni and Cu samples, but were still higher after the addition of urea to the electrolyte, which confirms its electrocatalytic activity toward urea oxidation.

To compare the activity of prepared layers potentials, a current density of 50 μA cm^−2^ was compared. In the 1 M KOH solution, only three samples, Ni I, Ni II and Cu I, showed a current response high enough to reach the boundary conditions. Among them, the Ni I sample showed the lowest potential needed for the desired current density, with a value of 0.793 V, while the Cu I sample showed the highest, with a value of 0.925 V. After the addition of urea to the electrolyte only the same three samples reached 50 μA cm^−2^. The lowest potential at which it was recorded was observed for the Ni II sample, with a value of 0.742 V. A very similar value of 0.752 V was observed for the Ni I sample, which, together with the higher activity of the Ni I sample in 1 M KOH solution, suggests that a higher content of nickel hydroxide in the PEO bath during the preparation of the Ni II sample does not necessary increase the electrocatalytic activity of the prepared layer toward the UOR. Interestingly, the Cu II sample showed a very similar activity, with a current of 50 μm cm^−2^ observed at a potential of 0.781 V.

The maximum current density observed for the Cu I sample at a potential of 1 V, was slightly lower than the values reported in the literature, i.e., for Ni deposited on an Fe_2_O_3_-modified TiO_2_ photocatalyst, which under dark conditions obtained a maximum value of 150 μA cm^−2^ [52]. At the same time, the current response of the Cu sample was twice as high as observed for a similar material prepared by covering the nickel foam with TiO_2_ paste [53]. However, the addition of nickel to the TiO_2_ system to form the photocatalyst for urea oxidation can lead to much higher current responses, such as the 360 μA cm^−2^ observed at 1.965 V [65] or the 10 mA cm^−2^ observed at 1.58 V [66], which shows that the activity of the prepared material can still be increased.

## 4. Conclusions

In the proposed research, an easy method of preparation of urea electrocatalysts was presented, using the plasma electrolytic oxidation of titanium in alkaline silicate solutions with Cu and Ni hydroxide suspensions. In the preliminary examinations, it has been proven that nickel and copper were incorporated into the growing oxide layer, resulting in a well-developed porous surface with a thickness greater than that reported for similar coating in the literature. The prepared surfaces were black, with a greenish appearance in the case of the nickel addition, and greyish one in the case of copper, which gives high chances of their potential photoactivity, which will be evaluated during further studies.

The addition of copper hydroxide to the electrolytic bath resulted in more hydrophilic layers. All layers enriched with Ni and Cu show electrochemical activity in 1 M KOH and in 1 M KOH + 0.15 M urea solutions, with the highest current response observed for the sample prepared in a Cu(OH)_2_ suspension, while the lowest potential needed for the 50 μA cm^−2^ was observed for the sample prepared in a Ni(OH)_2_ suspension. Such results are very promising, and a further increase in the electrocatalytic activity of oxide layers formed during PEO will be evaluated during further studies.

## Figures and Tables

**Table 1:** Sample code clarification and electrolytic bath compositions.

Sample	KOH, M	Na_2_SiO_3_, M	Ni(OH)_2_, M	Cu(OH)_2_, M	Voltage, V
Ref	0.05	0.5	-	-	200
Ni I			0.05	-	
Ni II			0.1	-	
Cu I			-	0.05	
Cu II			-	0.1	180

**Table 2:** Layer content in terms of atomic %, examined using EDX.

Sample		Ti	O *	Si	Na	K	Cu	Ni
Ref	Surface	2.1	70.6	21.1	5.7	0.6	-	-
	Cross section	9.1	77.7	11.7	1.4	-	-	-
Ni I	Surface	1.4	70.4	21.0	6.4	0.5	-	0.3
	Cross section	9.6	76.5	13.0	0.9	-	-	0.1
Ni II	Surface	1.7	70.5	20.0	6.9	0.6	-	0.3
	Cross section	10.13	77.3	11.6	0.8	-	-	0.1
Cu I	Surface	1.6	70.8	20.8	6.2	0.6	-	-
	Cross section	9.2	77.9	12.5	0.5	-	+	-
Cu II	Surface	2.1	70.8	19.8	6.8	0.6	+	-
	Cross section	9.2	78.1	11.9	0.6	-	0.1	-

* the values must be considered only as informative; “+”—elements were detected with a concentration below 0.3 at%.

**Table 3:** Comparison of the amount of Ni and Cu incorporated during PEO in various electrolytes.

Sample	PEO Bath	Form of Metal Additive	Metal Concentration, M	Metal Presence in the Layer, at% (EDS Data)	Reference
Cu I	Alkaline	Hydroxide suspension	0.05	0	This paper
Cu II			0.10	0	
Ni I			0.05	0.3	
Ni II			0.10	0.3	
Cu	Alkaline	Oxide	0.04	3.22 (wt%)	[18]
Ni	Neutral	Acetate	0.06	1.25	[26]
Ni	Neutral	Acetate	0.08	4.8 (XRD) 5.5 (XES)	[27,28,29]
Cu			0.025	0.4 (XRD) 5.9 (XES)	
Cu	Acidic	Nitrite	1.04	0.41	[32]
Cu	Acidic	Nitrite	2.70	2.59	[33]
Ni	Acidic	Acetate	0.05	4.3	[34]
Ni	Alkaline	Acetate	0.03	1.07	[30]

at%—atomic percentage; EDS—energy dispersive spectroscopy; wt%—weight percentage; XRD—X-ray diffraction analysis; XES—X-ray emission spectroscopy.

**Table 4:** Results of surface roughness analysis in terms of the *R_a_* and *R_z_* parameters.

Sample	Average *R_a_*, μm	Average *R_z_*, μm
Ref	5.43 ± 1.33	31.67 ± 6.53
Ni I	6.03 ± 0.75	33.89 ± 3.87
Ni II	6.25 ± 0.99	34.51 ± 3.35
Cu I	6.34 ± 0.77	34.37 ± 1.21
Cu II	5.52 ± 0.12	30.58 ± 2.56

**Table 5:** Comparison between current responses recorded in 1 M KOH and in 1 M KOH solutions and potentials needed for the current density of 50 μA cm^−2^ in 1 M KOH and 1 M KOH + 0.15 M urea solutions.

Sample	i@1 V, μA cm^−2^ in 1 M KOH	i@1 V, μA cm^−2^ in 1 M KOH + 0.15 M Urea	E@50 uA cm^−2^, V in 1 M KOH	E@50 μA cm^−2^, V in 1 M KOH + 0.15 M Urea
Ref	5	2	-	-
Ni I	99	102	0.793	0.752
Ni II	79	123	0.850	0.742
Cu I	71	130	0.925	0.781
Cu II	15	22	-	-

## Data Availability

Data available on request.
